# Supplementary material for: Academic case reports lack diversity: Assessing the presence and diversity of sociodemographic and behavioral factors related to Post COVID-19 Condition
Source: PLoS One. 2025 Jul 2;20(7):e0326668. doi: 10.1371/journal.pone.0326668 (PMC12221070; doi:10.1371/journal.pone.0326668)
Supplement: S1 Appendix A — S1 A.1 Additional explorations on the corpus with GPT. S1 A.2 Entity label refinement. S1 A.3 Sample prompt to GPT-4o produce variations per entity type. S1 A.4 Dictionary of variations per entity type (Produced by GPT-4o). S1 A.5 Sample prompt to generate sentence structures for augmentation of the development set. S1 A.6 Sample prompt to generate sentence structures for augmentation of the generalization set. S1 Appendix B. SDOH extraction and analysis pipeline details. In this section we provide additional information on the NER and NLI pipelines. S1 B.1 Sample prompts to GPT-4o for entailment and contradiction set generation. S1 B.2 NLI entailment and contradiction sets by entity type (generated by GPT-4o). S1 B.3 STable 1. Natural Language Inference (NLI) statements by entity type dimension. The statements were crafted to represent meaningful binary distinctions in the data. S1 B.5 STable 3. Performance of RNN and GRU models. S1 B.6 Fined-Tuned BERT-Base-Uncased Model Configuration. [file pone.0326668.s001.docx]

Supporting Information

**S1 Appendix A**. Corpus Construction Annotation Details

In this section, we provide further details on the entity annotation process for PCC case report corpus.

**S1 A.1** Additional explorations on the corpus with GPT

For additional robustness on the alignment with the extracted papers with long COVID, we experimented with prompting OpenAI’s GPT 3.5 Turbo API to classify whether on a subset of 2,000 papers were related to long COVID or not. As we found, however, the model had difficulty classifying the texts in a consistent and robust manner, as the definition of long COVID is highly dependent on the time of the first diagnosis with COVID-19 infection, which is rarely discussed in the case reports.

For the annotation task of subset 1, we explored three different techniques. Initially, we prompted the GPT-3.5 turbo (API) model to classify each token on the case report sections, based on a curated list of labels derived from those indicated by John Snow Labs, literature review and expert advice. Although we found that the model performed well with classification of a few entities, it did not perform well on a multi-class classification task of more than 10 labels.

**S1 A.2** Entity Label Refinement

To streamline the entity set, we consolidated closely related labels and removed those deemed non-essential for our use case. This process is outlined in sFigure 1 below.


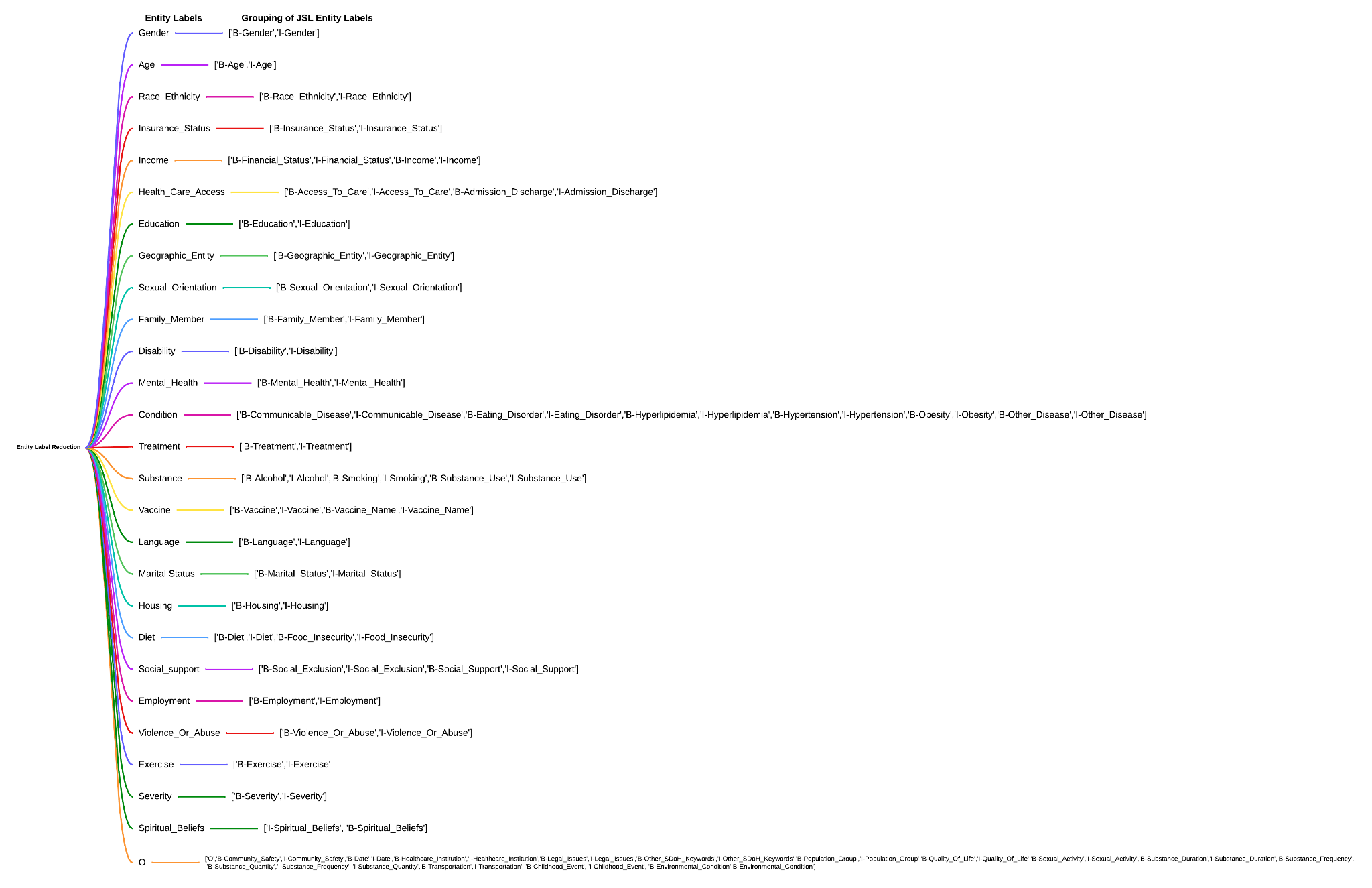


**S1 A.3** Sample prompt to GPT-4o to produce variations per entity type.

“For named entity recognition tasks using the CoNLL format, labels are split into B-label and I-label, where B signifies the beginning and I signifies the insight of a label for multi-word entities. Please provide two lists of diverse single-word variations related to this entity type dimension {label}, with this definition {definition}. One list should include words for B-label and the other list should be for words for I-label. Ensure that there is no overlap between the lists. Here is an example {example}”

**S1 A.4** Dictionary of Variations per Entity Type (Produced by GPT-4o).

variations\_rare\_entities=\{'B-Social\_Support':["community", "group", "peer", "mutual", "volunteer", "support", "counseling", "welfare", "social", "charity", "family", "neighborly", "emergency", "relief", "disaster"], 'I-Social\_Support':["service", "therapy", "assistance", "aid", "work", "group", "session", "service", "work", "organization", "support", "help", "shelter", "aid", "response"], 'B-Race\_Ethnicity':["Latino", "Black", "Asian", "White", "Hispanic", "Native", "African", "European", "Arab", "Pacific", "Indigenous", "Mixed", "Jewish", "Indian", "Korean"], 'I-Race\_Ethnicity':["American", "Canadian", "British", "Australian", "Islander", "Peoples", "Race", "Community", "Descent", "Ethnicity", "Heritage", "Culture", "Group", "Population", "Minority"],'B-Exercise':["running", "swimming", "cycling", "weightlifting", "yoga", "aerobics", "pilates", "hiking", "dancing", "jogging", "boxing", "rowing", "climbing", "jumping", "skipping"],'I-Exercise':["routine", "session", "workout", "practice", "training", "regimen", "activity", "drill", "movement", "exercise", "stretch", "program", "set", "circuit", "plan"], 'B-Education':["school", "college", "university", "academy", "institute", "kindergarten", "preschool", "highschool", "middle", "elementary", "primary", "secondary", "vocational", "training", "seminar"], 'I-Education':["degree", "diploma", "course", "program", "class", "lesson", "certificate", "studies", "education", "learning", "major", "specialization", "qualification", "curriculum", "instruction"], 'B-Geographic\_Entity':["Paris", "Canada", "Tokyo", "Brazil", "Berlin", "India", "London", "China", "Sydney", "Mexico", "Rome", "Russia", "Chicago", "Egypt", "Madrid"], 'I-Geographic\_Entity':["province", "capital", "metropolis", "country", "district", "suburb", "state", "township", "territory", "neighborhood", "island", "county", "territory", "zone", "area"], 'B-Substance':["alcohol", "cigarette", "marijuana", "cocaine", "heroin", "methamphetamine", "ecstasy", "LSD", "psilocybin", "ketamine", "opioid", "amphetamine", "tobacco", "caffeine", "nicotine"], 'I-Substance':["use", "consumption", "dependence", "addiction", "misuse", "habit", "intoxication", "ingestion", "administration", "dose", "exposure", "indulgence", "practice", "dependence"],'B-Severity':["severe", "critical", "extreme", "acute", "intense", "serious", "grave", "significant", "major", "urgent", "critical", "drastic", "dire", "severe", "crucial"], 'I-Severity':["condition", "case", "situation", "stage", "level", "instance", "circumstance", "state", "point", "stage", "level", "degree", "severity", "intensity", "extent"], 'B-Marital\_Status':["single", "married", "divorced", "widowed", "separated", "engaged", "partnered", "unmarried", "committed", "relationship", "civil", "union", "spouse", "marriage"], 'I-Marital\_Status':["status", "relationship"], 'B-Disability':["physical", "intellectual", "developmental", "mental", "emotional", "sensory", "cognitive", "psychological", "chronic", "visible", "invisible", "permanent", "temporary", "disability", "impairment"], 'I-Disability':["condition", "disorder", "issue", "challenge", "disability", "impairment", "problem", "limitation", "affliction", "ailment", "handicap", "constraint", "barrier", "hurdle", "obstacle"], 'B-Housing':["homeless", "renter", "homeowner", "tenant", "landlord", "lodger", "resident", "dweller", "occupant", "house", "apartment", "condominium", "townhouse", "villa", "shelter"], 'I-Housing':["living", "situation", "arrangement", "status", "condition", "accommodation", "housing", "unit", "space", "place", "option", "residence", "dwelling", "premises", "property"], 'B-Diet':["vegan", "vegetarian", "pescatarian", "carnivore", "omnivore", "keto", "paleo", "gluten-free", "low-carb", "low-fat", "high-protein", "balanced", "raw", "plant-based", "whole-food"], 'I-Diet':["diet", "plan", "regimen", "nutrition", "eating", "routine", "lifestyle", "habit", "practice", "approach", "program", "style", "protocol", "method", "choice"], 'B-Income':["income", "salary", "wage", "earnings", "pay", "compensation", "revenue", "stipend", "remuneration", "profit", "compensation", "recompense", "gain", "return", "receipt"], 'I-Income':["level", "bracket", "range", "category", "tier", "class", "scale", "segment", "division", "section", "grade", "classification", "band", "division", "stratum"], 'B-Language':["english", "spanish", "french", "mandarin", "arabic", "hindi", "bengali", "portuguese", "russian", "japanese", "german", "korean", "italian", "turkish", "vietnamese"], 'I-Language':["language", "dialect", "vernacular", "tongue", "speech", "communication", "idiom", "lingo", "jargon", "vocabulary", "lexicon", "phraseology", "linguistics", "grammar", "accent"], 'B-Insurance\_Status':["insured", "uninsured", "covered", "protected", "policyholder", "enrolled", "subscriber", "beneficiary", "underwritten", "guaranteed", "assured", "insurer", "coverage", "insurance", "plan"], 'I-Insurance\_Status':["status", "holder", "member", "recipient", "participating", "enrollee", "policy", "participant", "benefit", "coverage", "plan", "option", "program", "scheme", "arrangement"], 'B-Violence\_Or\_Abuse':["violence", "abuse", "assault", "battery", "harassment", "bullying", "intimidation", "coercion", "exploitation", "neglect", "trauma", "maltreatment", "domestic", "partner", "stalking"], 'I-Violence\_Or\_Abuse':["incident", "case", "act", "behavior", "occurrence", "confrontation", "abuse", "encounter", "occurrence"], 'B-Family\_Member':["father", "mother", "brother", "sister", "grandfather", "grandmother", "uncle", "aunt", "cousin", "son", "daughter", "nephew", "niece", "husband", "wife"], 'I-Family\_Member':["parent", "sibling", "grandparent", "relative", "family", "member", "child", "offspring", "kin", "spouse", "partner", "relation", "in-law", "step-", "adoptive"], 'B-Sexual\_Orientation':["gay", "lesbian", "bisexual", "heterosexual", "queer", "pansexual", "asexual", "demisexual", "homosexual", "biromantic", "heteroromantic", "sapiosexual", "aromantic", "bicurious", "questioning"], 'I-Sexual\_Orientation':["orientation", "identity", "preference", "inclination", "attraction", "orientation", "orientation", "identity", "orientation", "identity", "preference", "orientation", "identity", "orientation", "identity"], 'B-Gender':["man", "woman", "male", "female", "boy", "girl", "transgender", "nonbinary", "agender", "bigender", "cisgender", "genderfluid", "two-spirit"], 'I-Gender':["identity", "identifying"], 'B-Spiritual\_Beliefs':["christian", "islamic", "buddhist", "hindu", "jewish", "sikh", "taoist", "shinto", "bahai", "jain", "pagan", "wiccan", "new\_age", "atheist", "agnostic"], 'I-Spiritual\_Beliefs':["belief", "faith", "religion", "spirituality", "doctrine", "philosophy", "creed", "teaching", "tenet", "principle", "ideology", "conviction", "dogma", "mythology", "doctrine"],'B-Age' : ["young", "adult", "elderly", "child", "teenage", "middle-aged", "senior", "adolescent", "infant", "toddler", "youthful", "mature", "50-year-old", "30-year-old", "20-year-old", "10-year-old", "newborn"],'I-Age': ["years", "old", "age"],'B-Employment': ["employed", "unemployed", "worker", "employee", "jobholder", "laborer", "professional", "executive", "manager", "entrepreneur", "freelancer", "contractor", "temp", "part-time", "full-time", "doctor", "teacher", "engineer", "artist", "chef"], 'I-Employment': ["position", "role", "occupation", "job", "career", "work"], 'B-Vaccine': ["pfizer", "moderna", "astrazeneca", "johnson\_and\_johnson", "sinovac", "sinopharm", "sputnik", "vaccinated", "unvaccinated", "immunized"], 'I-Vaccine': ["vaccination", "immunization", "dose", "inoculation","vaccine"], 'B-Mental\_Health': ["depression", "anxiety", "bipolar", "schizophrenia", "ptsd", "OCD", "psychosis", "happiness", "self-care", "mindfulness", "psychiatric", "therapy"], 'I-Mental\_Health': ["state"], 'B-Treatment':["chemotherapy", "radiation", "dialysis", "transplant", "vaccination", "immunotherapy", "antibiotics", "antivirals", "physiotherapy", "chiropractic", "acupuncture", "orthopedic", "dermatological", "cardiac", "oncological", "surgery", "rehabilitation", "intervention", "treatment", "holistic", "palliative", "homeopathy"], 'I-Treatment': ["plan", "session", "regimen", "protocol", "procedure", "approach", "method", "intervention", "strategy", "technique", "therapy"], 'B-Access\_To\_Care': ["availability", "proximity", "affordability", "coverage", "accessibility", "transportation", "providers", "appointments", "clinics", "hospitals", "facilities", "ICU", "telehealth"],'I-Access\_To\_Care' :[ "barrier", "waitlist", "delay", "shortage", "gap", "disparity", "limitation", "restriction", "obstacle", "constraint"],"B-Condition":["asthma", "diabetes", "hypertension", "arthritis", "cancer", "stroke", "epilepsy", "dementia", "migraine", "cardiomyopathy", "anemia", "osteoporosis", "glaucoma", "parkinson"],"I-Condition":["diagnosis", "stage", "symptom", "manifestation", "prognosis", "complication", "episode", "onset", "recurrence", "chronic", "persistent", "latent", "remission"]\}

**S1 A.5** Sample prompt to generate sentence structures for augmentation of the development set.\\

“For a named entity recognition task using the CoNLL format, the following labels are utilized \{set\_of\_non\_o\_labels\}. Here are the definitions of each label dimension \{definitions\}.

Please generate three distinct sentence structures using all the provided labels once (in total). They should follow these conditions:

1) Labels being related to same entity dimension must follow each other in the order B-label I-label

2) Each sentence structure must have a different context and label order than the others

3) All spaces where the labels would occur are maintained with the label names

4) All sentences should include a minimal number of words outside of the labels, while maintaining contextual integrity

Here is an example: \{example\}”

**S1 A.6** Sample prompt to generate sentence structures for augmentation of the generalization set.

“For a named entity recognition task using the CoNLL format, the following labels are utilized \{set\_of\_non\_o\_labels\}. Here are the definitions of each label dimension \{definitions\}.

Please generate a set of three distinct sentence structures using all the provided labels once (in total). They should follow these conditions:

1) Labels being related to same entity dimension must follow each other in the order B-label I-label

2) Each sentence structure must have a different context and label order than the others

3) All spaces where the labels would occur are maintained with the label names

4) All sentence structures should have a distinct label order and a larger number of words than these sentence structures: \{previous\_sentence\_structures\}

Here is an example: \{example\}”

S1 Appendix B. SDOH Extraction and Analysis Pipeline Details.

In this section we provide additional information on the NER and NLI pipelines.

**S1 B.1** Sample Prompts to GPT-4o for entailment and contradiction set generation

“Please provide a list of all single words related to this \{entity\_label\_class\} that make the following statement true: \{Statement\}”

“Now please provide a list of all single words related to this \{entity\_label\_class\} that are contradictory the following statement: \{Statement\}”

**S1 B.2** NLI entailment and contradiction sets by entity type (generated by GPT-4o)

"Access To Care":{"entailment:[

"clinic",

"hospital",

"doctor",

"nurse",

"appointment",

"treatment",

"medication",

"pharmacy",

"insurance",

"provider",

"specialist",

"diagnosis",

"screening",

"therapy",

"consultation",

"referral",

"prescription",

"coverage",

"caregiver",

"facility",

"emergency",

"service",

"healthcare",

"benefit",

"ICU",

"intake",

"admitted",

"scheduled",

"enter"

]}, 'contradiction':[

"barrier",

"delay",

"unavailable",

"uninsured",

"inaccessible",

"cost",

"expense",

"inequality",

"disparity",

"distance",

"waitlist",

"overcrowding",

"outreach",

"underserved",

"limited",

"restriction",

"shortage",

"neglect",

"exclusion",

"inequity"

]},

'Age':{'entailment':['65',

'66',

'67',

'68',

'69',

'70',

'71',

'72',

'73',

'74',

'75',

'76',

'77',

'78',

'79',

'80',

'81',

'82',

'83',

'84',

'85',

'86',

'87',

'88',

'89',

'90',

'91',

'92',

'93',

'94',

'95',

'96',

'97',

'98',

'99',

'100',

'101',

'102',

'103',

'104',

'105',

'106',

'107',

'108',

'109',

'110',

'111',

'112',

'113',

'114',

'115',

'116',

'117',

'118',

'119',

'senior',

'elderly',

'aged',

'senior citizen',

'retiree',

'geriatric',

'old',

'mature',

'veteran',

'ancient',

'pensioner',

'octogenarian',

'nonagenarian',

'centenarian'], 'contradiction':['1',

'2',

'3',

'4',

'5',

'6',

'7',

'8',

'9',

'10',

'11',

'12',

'13',

'14',

'15',

'16',

'17',

'18',

'19',

'20',

'21',

'22',

'23',

'24',

'25',

'26',

'27',

'28',

'29',

'30',

'31',

'32',

'33',

'34',

'35',

'36',

'37',

'38',

'39',

'40',

'41',

'42',

'43',

'44',

'45',

'46',

'47',

'48',

'49',

'50',

'51',

'52',

'53',

'54',

'55',

'56',

'57',

'58',

'59',

'60',

'61',

'62',

'63',

'young',

'youthful',

'adolescent',

'teenager',

'juvenile',

'minor',

'child',

'preteen',

'teen',

'toddler',

'infant',

'preschooler',

'youngster',

'adult',

'youth',

'preadult',

'earlyadult']},

'Condition':{'entailment':[

"cancer",

"diabetes",

"fibrosis",

"Huntington",

"Parkinson",

"ALS",

"dementia",

"epilepsy",

"HIV",

"AIDS",

"lupus",

"sarcoma",

"leukemia",

"lymphoma",

"hemophilia",

"celiac",

"Crohn",

"colitis",

"sclerosis",

"malaria",

"tuberculosis",

"Alzheimer",

"thalassemia",

"cystinosis",

"marfan",

"sickle",

"lyme",

"endometriosis",

"myeloma",

"amyloidosis",

"neurofibromatosis",

"phenylketonuria",

"porphyria",

"retinoblastoma",

"scleroderma",

"spina",

"muscular",

"dystrophy",

"hypertension",

"cardiomyopathy",

"hepatitis",

"cirrhosis",

"nephropathy",

"uremia",

"glioblastoma"

], 'contradiction':[

"headache",

"fever",

"cough",

"nausea",

"fatigue",

"dizziness",

"sore",

"itching",

"rash",

"cold",

"flu",

"allergy",

"sprain",

"bruise",

"infection",

"constipation",

"diarrhea",

"vomiting",

"stomachache",

"indigestion",

"burn",

"cut",

"blister",

"acne",

"cramp",

"fracture",

"swelling",

"nosebleed",

"sneezing",

"hiccup",

"thirst",

"dehydration",

"insomnia",

"anxiety",

"stress",

"tremor",

"irritation",

"dryness",

"fainting",

"bloating",

"heartburn",

"itchiness",

"runny",

"congestion",

"sensitivity",

"numbness"

]},

'Diet':{'entailment':[

"vegan",

"vegetarian",

"paleo",

"keto",

"gluten-free",

"dairy-free",

"halal",

"kosher",

"pescatarian",

"low-carb",

"low-fat",

"low-sodium",

"mediterranean",

"carnivore",

"macrobiotic",

"whole30",

"alkaline",

"raw",

"frugivore",

"fasting"

], 'contradiction':[

"omnivore",

"balanced",

"flexitarian",

"standard",

"mixed",

"unrestricted",

"traditional",

"casual",

"regular",

"moderate"

]},

'Disability':{'entailment':[

"disabled",

"person-with-disability",

"individual-with-disability",

"differently-abled",

"challenged",

"handicapped",

"impaired",

"physically-disabled",

"mentally-disabled",

"visual-impairment",

"hearing-impairment",

"deaf",

"blind",

"partially-sighted",

"low-vision",

"mobility-impairment",

"wheelchair-user",

"spinal-cord-injury",

"amputee",

"cerebral-palsy",

"multiple-sclerosis",

"muscular-dystrophy",

"autism-spectrum-disorder",

"ADHD",

"attention-deficit-disorder",

"intellectual-disability",

"developmental-disability",

"epilepsy",

"chronic-pain",

"chronic-fatigue-syndrome",

"fibromyalgia",

"mental-health-condition",

"bipolar-disorder",

"schizophrenia",

"depression",

"anxiety-disorder",

"PTSD",

"post-traumatic-stress-disorder"

], "contradiction":[

"able-bodied",

"able",

"without-disability",

"fully-abled",

"healthy",

"functioning-normally",

"typically-abled",

"normal",

"intact",

"capable",

"unrestricted",

"unaffected",

"well",

"fit",

"active",

"independent",

"non-impaired",

"unrestricted-functioning",

"without-condition",

"optimal-health",

"normal-functioning"]},

"Education":{"entailment":[

"alumni",

"assignment",

"bachelor",

"capstone",

"credit",

"diploma",

"dissertation",

"enrollment",

"graduation",

"honors",

"internship",

"major",

"master's",

"prerequisite",

"research",

"semester",

"sophomore",

"thesis",

"transcript",

"undergraduate",

"university",

"vocational",

"doctor",

"engineer",

"nurse",

"specialist",

"GED",

"lawyer"

], "contradiction":[

"basic",

"classroom",

"concept",

"elementary",

"exercise",

"homework",

"schoolwork",

"young",

"child",

"infant",

"unskilled",

"illiterate",

"middleschool",

"kindergarten",

"baby",

"childcare",

"play",

"uneducated",

"alphabet",

"book",

"class",

"coloring",

"daily",

"drawing",

"interactive",

"literacy",

"map",

"quiz"

]},

"Employment":{"entailment":[

"accountant",

"application",

"benefits",

"boss",

"career",

"contract",

"coworker",

"daycare",

"employee",

"employer",

"engineer",

"hire",

"intern",

"job",

"manager",

"nurse",

"office",

"payroll",

"position",

"promotion",

"recruiter",

"resume",

"salary",

"secretary",

"staff",

"supervisor",

"task",

"team",

"technician",

"trainer",

"vacation",

"wage",

"doctor",

"nurse"

], "contradiction":[

"absence",

"benefits",

"crisis",

"downtime",

"furlough",

"jobless",

"layoff",

"loss",

"out-of-work",

"recession",

"redundancy",

"resignation",

"severance",

"situation",

"suspension",

"termination",

"unemployed",

"vacant",

"welfare",

"without",

"retraining",

"searching",

"displacement"

]},

"Exercise":{"entailment":[

"Cardio",

"Jogging",

"Cycling",

"Swimming",

"Rowing",

"CrossFit",

"Lifting",

"Pilates",

"Yoga",

"Running",

"Drills",

"Training",

"Conditioning",

"Workouts",

"Mobility",

"Aerobics"

], "contradiction":[

"Sedentary",

"Idle",

"Inactive",

"Dormant",

"Lazy",

"Unfit",

"Neglect",

"Lethargic",

"Sluggish",

"Unmotivated",

"Resting",

"Indolent",

"Procrastination",

"Apathetic",

"Static",

"Stagnant",

"Leisurely",

"Avoidance",

"Neglectful",

"Out-of-shape"

]},

"Family Member":{"entailment":[

"child",

"son",

"daughter"

], "contradiction":[

"cousin",

"sister",

"brother",

"sibling",

"grandma",

"grandpa"

]},

"Gender":{"entailment":[

"Woman",

"Female",

"Girl",

"Lady",

"Wife",

"Daughter",

"Gal",

"Miss",

"Sister",

"Queen",

"Matriarch",

"Mother",

"Diva",

"Chick",

"Ma'am",

"she",

"girl",

"grandmother",

"feminine",

"sister"

], "contradiction":[

"Man",

"Male",

"Boy",

"Gentleman",

"Husband",

"Son",

"Dude",

"Guy",

"Brother",

"Patriarch",

"Father",

"Macho",

"he",

"Nonbinary",

"Genderqueer",

"Genderfluid",

"Agender",

"Bigender",

"Demiboy"

]},

"Geographic Entity":{"entailment":[

"England",

"Scotland",

"Wales",

"Ireland",

"USA",

"Canada",

"Australia",

"New Zealand",

"London",

"Sydney",

"Toronto",

"Dublin",

"Edinburgh",

"Melbourne",

"Vancouver",

"Glasgow",

"Auckland",

"Houston",

"Chicago",

"San Francisco",

"New York",

"Los Angeles",

"Boston",

"Seattle",

"Brisbane",

"Perth",

"Calgary",

"Ottawa",

"Manchester",

"Belfast",

"Wellington",

"Adelaide",

"Kansas City",

"Philadelphia",

"Atlanta",

"San Diego",

"Minneapolis",

"Baltimore",

"United States",

"New York",

"State",

"Province"], "contradiction":[

"France",

"Germany",

"Spain",

"Italy",

"China",

"Japan",

"Russia",

"Brazil",

"South Korea",

"Mexico",

"India",

"Argentina",

"Turkey",

"Saudi Arabia",

"Thailand",

"Sweden",

"Norway",

"Denmark",

"Greece",

"Netherlands",

"Portugal",

"Poland",

"Czechia",

"Hungary",

"Vietnam",

"Malaysia",

"Indonesia",

"Egypt",

"Chile",

"Colombia",

"Iran",

"Israel",

"Pakistan",

"Bangladesh",

"Ukraine",

"Peru",

"Romania",

"Jordan",

"Philippines",

"Sri Lanka",

"Nepal",

"UAE"

]},

"Housing":{"entailment":[

"Homeless",

"Displaced",

"Vagrant",

"Transient",

"Squatter",

"Houseless",

"Destitute",

"Unhoused",

"Wanderer",

"Nomadic",

"Refugee",

"Drifter",

"Impoverished",

"Indigent",

"Evicted",

"Exiled",

"Unsheltered",

"Rootless",

"Vagabond",

"Outcast",

"Shelter",

"Temporary"

], "contradiction":[

"Housed",

"Settled",

"Lodged",

"Dweller",

"Tenant",

"Homeowner",

"Proprietor",

"Lodger",

"Boarder",

"Leaseholder",

"Renter",

"Landlord",

"Landlady",

"Householder",

"Proprietor",

"house",

"apartment",

"condo",

"villa",

"cottage"

]},

"Income":{"entailment":[

"high-income",

"Wealthy",

"Affluent",

"Prosperous",

"Rich",

"Well-off",

"Loaded",

"Flourishing",

"Opulent",

"Lavish",

"Thriving",

"Comfortable",

"Elite",

"Privileged",

"Millionaire",

"Billionaire",

"Affluence",

"Tycoon",

"Magnate",

"Aristocrat",

"Plutocrat"

], "contradiction":[

"Poor",

"Impoverished",

"Needy",

"Struggling",

"Destitute",

"Low-income",

"Underprivileged",

"Broke",

"Indigent",

"Disadvantaged",

"Penniless",

"Hard-up",

"Hand-to-mouth",

"Economizing",

"Frugal",

"Working-class",

"Modest",

"Income-restricted",

"Underpaid",

"Subsidized"

]},

"Insurance Status":{"entailment":[

"insured",

"covered",

"protected",

"policyholder",

"subscriber",

"beneficiary",

"enrolled",

"planholder",

"participant",

"member",

"certificate-holder",

"dependent",

"claimant",

"premium-payer",

"co-insured",

"beneficiary-owner",

"enrollee",

"recipient",

"policy-beneficiary",

"co-pay-holder"

], "contradiction":[

"uninsured",

"unprotected",

"uncovered",

"self-pay",

"out-of-pocket",

"non-covered",

"non-insured",

"underinsured",

"vulnerable",

"unsubscribed",

"unregistered",

"non-enrolled",

"policy-lapsed",

"unaffiliated",

"non-member",

"not-covered",

"excluded",

"no-coverage"

]},

"Language":{"entailment":[

"anglophone",

"english-speaking",

"english-talking",

"english-fluent",

"english-proficient",

"english-literate",

"english-user",

"english-native",

"english-conversant",

"english-articulate",

"english-communicator",

"english",

"biligual"

], "contradiction":[

"spanish",

"french",

"german",

"mandarin",

"arabic",

"russian",

"hindi",

"japanese",

"korean",

"italian",

"portuguese",

"swahili",

"urdu",

"bengali",

"vietnamese",

"turkish",

"thai",

"persian",

"polish",

"dutch",

"greek",

"hebrew",

"malay",

"tagalog",

"indonesian",

"tamil",

"telugu",

"swedish",

"norwegian",

"finnish",

"danish",

"punjabi",

"gujarati",

"cantonese",

"ukrainian",

"hungarian",

"romanian",

"czech",

"slovak",

"serbian",

"croatian",

"bosnian",

"bulgarian",

"albanian",

"georgian",

"armenian",

"kurdish",

"pashto",

"somali",

"zulu",

"amharic",

"yiddish"

]},

"Marital Status":{"entailment":[

"married",

"wed",

"spouse",

"husband",

"wife",

"partnered",

"wedded",

"hitched",

"betrothed",

"matrimonial",

"nuptial",

"coupled",

"joined",

"in-union",

"espoused",

"conjugally-bound",

"life-partner",

"in-wedlock",

"legally-bound",

"spousal"

], "contradiction":[

"unmarried",

"single",

"bachelor",

"bachelorette",

"divorced",

"widowed",

"separated",

"not-in-union",

"unwed",

"not-married",

"solo",

"independent",

"available",

"unpartnered",

"single-status",

"unconjugated",

"not-attached",

"non-marital",

"single-person",

"uncommitted"

]},

"Mental Health":{"entailment":[

"anxiety-disorder",

"depression",

"bipolar-disorder",

"schizophrenia",

"obsessive-compulsive-disorder",

"post-traumatic-stress-disorder",

"attention-deficit-hyperactivity-disorder",

"borderline-personality-disorder",

"eating-disorder",

"panic-disorder",

"social-anxiety-disorder",

"generalized-anxiety-disorder",

"schizoaffective-disorder",

"autism-spectrum-disorder",

"dysthymia",

"seasonal-affective-disorder",

"psychotic-disorder",

"somatic-symptom-disorder",

"dissociative-identity-disorder",

"paranoid-personality-disorder",

"narcissistic-personality-disorder",

"avoidant-personality-disorder",

"dependent-personality-disorder",

"histrionic-personality-disorder",

"substance-use-disorder",

"sleep-disorder",

"bipolar-II-disorder",

"premenstrual-dysphoric-disorder",

"trichotillomania",

"hoarding-disorder",

"impulse-control-disorder",

"delusional-disorder",

"antisocial-personality-disorder",

"psychotic-break",

"complex-post-traumatic-stress-disorder",

"neurocognitive-disorder",

"paranoid-schizophrenia",

"schizophreniform-disorder",

"reactive-attachment-disorder",

"selective-mutism",

"gender-dysphoria"

], "contradiction":[

"emotional-regulation",

"self-care",

"mindfulness",

"emotional-health",

"self-esteem",

"mental-fitness",

"meditation",

"self-awareness",

"adjustment",

"relaxation",

"emotional-support",

"mental-clarity",

"behavioral-health",

"personal-growth",

"emotional-resilience",

"stress-management",

"mental-state",

"emotional-wellness",

"life-skills",

"self-help",

"emotional-intelligence",

"wellness",

"positive-mental-health"

]},

"Race Ethnicity":{"entailment":[

"white",

"caucasian",

"european",

"anglo",

"non-hispanic-white",

"of-european-descent",

"light-skinned",

"fair-skinned",

"euro-american",

"euro-descendant",

"western-european",

"ethnically-white",

"white-american",

"white-european",

"caucasoid",

"british",

"irish",

"german",

"french",

"italian",

"spanish",

"portuguese",

"scandinavian",

"dutch",

"belgian",

"austrian",

"swiss",

"polish",

"hungarian",

"romanian",

"ukrainian",

"greek",

"bulgarian",

"serbian",

"croatian",

"slovak",

"czech",

"slovenian",

"estonian",

"latvian",

"lithuanian",

"russian",

"georgian",

"armenian"

], "contradiction":[

"black",

"african-american",

"african",

"hispanic",

"latino",

"latina",

"asian",

"pacific-islander",

"native-american",

"indigenous",

"middle-eastern",

"arab",

"south-asian",

"east-asian",

"southeast-asian",

"native-hawaiian",

"american-indian",

"alaskan-native",

"mixed-race",

"biracial",

"multiracial",

"indian",

"pakistani",

"bangladeshi",

"nepalese",

"tibetan",

"mongolian",

"samoan",

"tongan",

"fijian",

"maori",

"aboriginal",

"torres-strait-islander",

"latinx",

"nigerian",

"kenyan",

"ghanaian",

"south-african",

"jamaican",

"brazilian",

"colombian",

"argentinian",

"peruvian",

"chilean",

"japanese",

"south-korean",

"vietnamese",

"filipino",

"myanmar",

"laotian",

"cambodian",

"fijian",

"papuan",

"aboriginal-australian",

"torres-strait-islander",

"saudi-arabian",

"iranian",

"iraqi",

"jordanian",

"egyptian",

"lebanese",

"syrian",

'chi',

'chin',

'chun',

'chung',

'hong',

'jia',

'joo',

'ko',

'kong',

'ku',

'wang',

'wet',

'wong',

'wu',

'xu',

'yue'

]},

"Severity":{"entailment":[

"severe",

"acute",

"critical",

"life-threatening",

"grave",

"serious",

"debilitating",

"intense",

"extreme",

"advanced",

"terminal",

"dangerous",

"worsening",

"incapacitating",

"drastic",

"progressive",

"persistent",

"unmanageable",

"high-risk",

"fatal",

"irreversible",

"end-stage",

"excruciating",

"complicated",

"intractable",

"malignant",

"emergency",

"unstable",

"alarming",

"deteriorating",

"profound"

], "contradiction":[

"mild",

"minor",

"moderate",

"manageable",

"low-risk",

"non-serious",

"stable",

"temporary",

"recoverable",

"benign",

"treatable",

"contained",

"localized",

"slight",

"limited",

"minimal",

"controlled",

"subtle",

"non-life-threatening",

"infrequent",

"improving",

"marginal",

"relieving",

"reversible",

"light"

]},

"Sexual Orientation":{"entailment":[

"heterosexual",

"straight",

"opposite-sex-attracted",

"hetero",

"male-female",

"heteronormative",

"binary-attracted",

"heterosexual-couple",

"man-woman-relationship",

"traditional-relationship",

"hetero-cisgender"

], "contradiction":[

"homosexual",

"gay",

"lesbian",

"bisexual",

"pansexual",

"queer",

"asexual",

"demisexual",

"polysexual",

"omnisexual",

"non-binary-attracted",

"same-sex-attracted",

"genderqueer-attracted",

"fluid-sexuality",

"two-spirit",

"androgynous-attracted",

"sapphic",

"achillean"

]},

"Social Support":{"entailment":[

"support",

"help",

"assistance",

"guidance",

"community",

"network",

"companionship",

"friendship",

"family-support",

"emotional-support",

"counsel",

"peer-support",

"mentorship",

"encouragement",

"aid",

"backup",

"care",

"nurturing",

"reassurance",

"solidarity",

"comfort",

"advocacy",

"protection",

"cooperation",

"teamwork",

"social-network",

"collaboration",

"partnership",

"shared-responsibility",

"associates",

"alliance"

], "contradiction":[

"isolation",

"loneliness",

"neglect",

"abandonment",

"alienation",

"disconnection",

"estrangement",

"solitude",

"detachment",

"lack-of-support",

"unsupported",

"unassisted",

"neglected",

"forsaken",

"social-exclusion",

"marginalization",

"rejection",

"disassociation",

"disengagement",

"seclusion",

"friendless",

"abandoned",

"unhelped",

"solitary",

"outcast",

"unprotected",

"uncared-for",

"disempowered",

"isolated",

"unsupported"

]},

"Spiritual Beliefs":{"entailment":[

"christian",

"catholic",

"christianity",

"roman-catholic",

"baptist",

"protestant",

"evangelical",

"methodist",

"presbyterian",

"orthodox-christian",

"lutheran",

"anglican",

"episcopalian",

"born-again",

"follower-of-christ",

"believer",

"churchgoer",

"disciple",

"catholicism",

"christ-follower",

"christendom",

"clergy",

"parishioner",

"faithful",

"apostolic",

"orthodox",

"jesus-follower",

"christian-believer"

], "contradiction":[

"islam",

"muslim",

"judaism",

"jewish",

"hindu",

"hinduism",

"buddhism",

"buddhist",

"sikhism",

"sikh",

"jainism",

"jain",

"taoism",

"taoist",

"shinto",

"shintoism",

"confucianism",

"zoroastrianism",

"zoroastrian",

"pagan",

"wiccan",

"animism",

"bahá'í",

"rastafarian",

"indigenous-religions",

"shamanism",

"druze",

"bahá'í-faith",

"neo-pagan",

"voodoo",

"santeria"

]},

"Substance":{"entailment":[

"cannabis",

"marijuana",

"THC",

"cocaine",

"heroin",

"LSD",

"acid",

"psilocybin",

"magic-mushrooms",

"MDMA",

"ecstasy",

"molly",

"methamphetamine",

"meth",

"amphetamine",

"adderall",

"methylphenidate",

"ritalin",

"ketamine",

"PCP",

"phencyclidine",

"mescaline",

"peyote",

"ayahuasca",

"DMT",

"salvia",

"opium",

"morphine",

"oxycodone",

"oxycotin",

"fentanyl",

"benzodiazepines",

"xanax",

"valium",

"alcohol",

"nicotine",

"tobacco",

"caffeine",

"kratom",

"synthetic-cannabinoids",

"bath-salts",

"inhalants",

"cigarretes",

"alcohol"

], "contradiction":[

"sober",

"abstinent",

"clean",

"drug-free",

"substance-free",

"non-user",

"teetotaler",

"in-recovery",

"rehabilitated",

"non-smoking",

"alcohol-free",

"straight-edge",

"clean-living",

"addiction-free",

"detoxified",

"dry",

"temperate",

"non-dependent",

"no-drug-use",

"no-substance-use"

]},

"Treatment":{"entailment":[

"surgery",

"surgical-procedure",

"operation",

"open-surgery",

"organ-transplant",

"bone-marrow-transplant",

"invasive-procedure",

"chemotherapy",

"radiotherapy",

"radiation-therapy",

"neurosurgery",

"laparotomy",

"thoracotomy",

"ablation",

"hysterectomy",

"gastrectomy",

"amputation",

"percutaneous",

"endoscopic-surgery",

"stent-placement",

"intravenous-catheter",

"peritoneal-dialysis",

"cardiac-catherization",

"implants",

"artificial-heart",

"ventricular-assist-device"

], "contradiction":[

"medication",

"pharmacotherapy",

"physical-therapy",

"occupational-therapy",

"psychotherapy",

"counseling",

"acupuncture",

"massage-therapy",

"chiropractic",

"herbal-remedies",

"homeopathy",

"dietary-changes",

"exercise",

"lifestyle-modification",

"biofeedback",

"light-therapy",

"transcutaneous-electrical-nerve-stimulation",

"TENS",

"speech-therapy",

"rehabilitation",

"orthotics",

"support-braces",

"cold-therapy",

"heat-therapy",

"hydrotherapy",

"ultrasound-therapy"

]},

"Vaccine":{"entailment":[

"vaccinated",

"fully-vaccinated",

"COVID-19-vaccination",

"COVID-vaccine",

"immunized",

"COVID-immunization",

"COVID-shot",

"COVID-injection",

"Pfizer-vaccine",

"Comirnaty",

"Moderna-vaccine",

"Spikevax",

"JohnsonandJohnson-vaccine",

"Janssen-vaccine",

"AstraZeneca-vaccine",

"Vaxzevria",

"Sinopharm-vaccine",

"Sinovac-vaccine",

"CoronaVac",

"Covaxin",

"Covovax",

"Novavax-vaccine",

"COVID-booster-shot",

"booster-dose",

"COVID-19-vaccinated",

"immunized-against-COVID",

"COVID-protection",

"pandemic-vaccine"

], "contradiction":[

"unvaccinated",

"not-vaccinated",

"COVID-19-unvaccinated",

"COVID-unimmunized",

"not-immunized",

"without-COVID-vaccine",

"vaccine-free",

"vaccine-resistant",

"vaccine-averse",

"COVID-19-non-vaccinated",

"unimmunized",

"vaccine-refusal",

"unprotected",

"not-inoculated",

"without-COVID-shot",

"without-COVID-injection",

"vaccine-exempt",

"non-vaccinated",

"not-COVID-vaccinated",

"vaccine-non-compliant"

]},

"Violence Or Abuse":{"entailment":[

"abused",

"victim-of-violence",

"survivor-of-abuse",

"trauma-survivor",

"domestic-violence-victim",

"sexually-assaulted",

"physically-assaulted",

"emotionally-abused",

"neglected",

"exploited",

"harassed",

"battered",

"maltreated",

"beaten",

"victim-of-trauma",

"suffered-abuse",

"experienced-violence",

"subjected-to-abuse",

"abuse-victim",

"assaulted",

"intimidated",

"bullied",

"coerced",

"manipulated",

"emotionally-traumatized",

"psychologically-abused",

"financially-exploited",

"sexually-exploited"

], "contradiction":[

"unaffected",

"free-from-abuse",

"abuse-free",

"trauma-free",

"safe",

"secure",

"protected",

"well-adjusted",

"healthy",

"stable",

"non-victim",

"resilient",

"not-experienced-abuse",

"not-subjected-to-violence",

"emotionally-healthy",

"unharmed",

"not-exposed-to-abuse",

"not-assaulted",

"not-experienced-trauma",

"non-traumatized",

"non-exploited",

"well-supported",

"non-victimized"

]}

**S1 B.3** STable 1. Natural Language Inference (NLI) statements by entity type dimension. The statements were crafted to represent meaningful binary distinctions in the data.


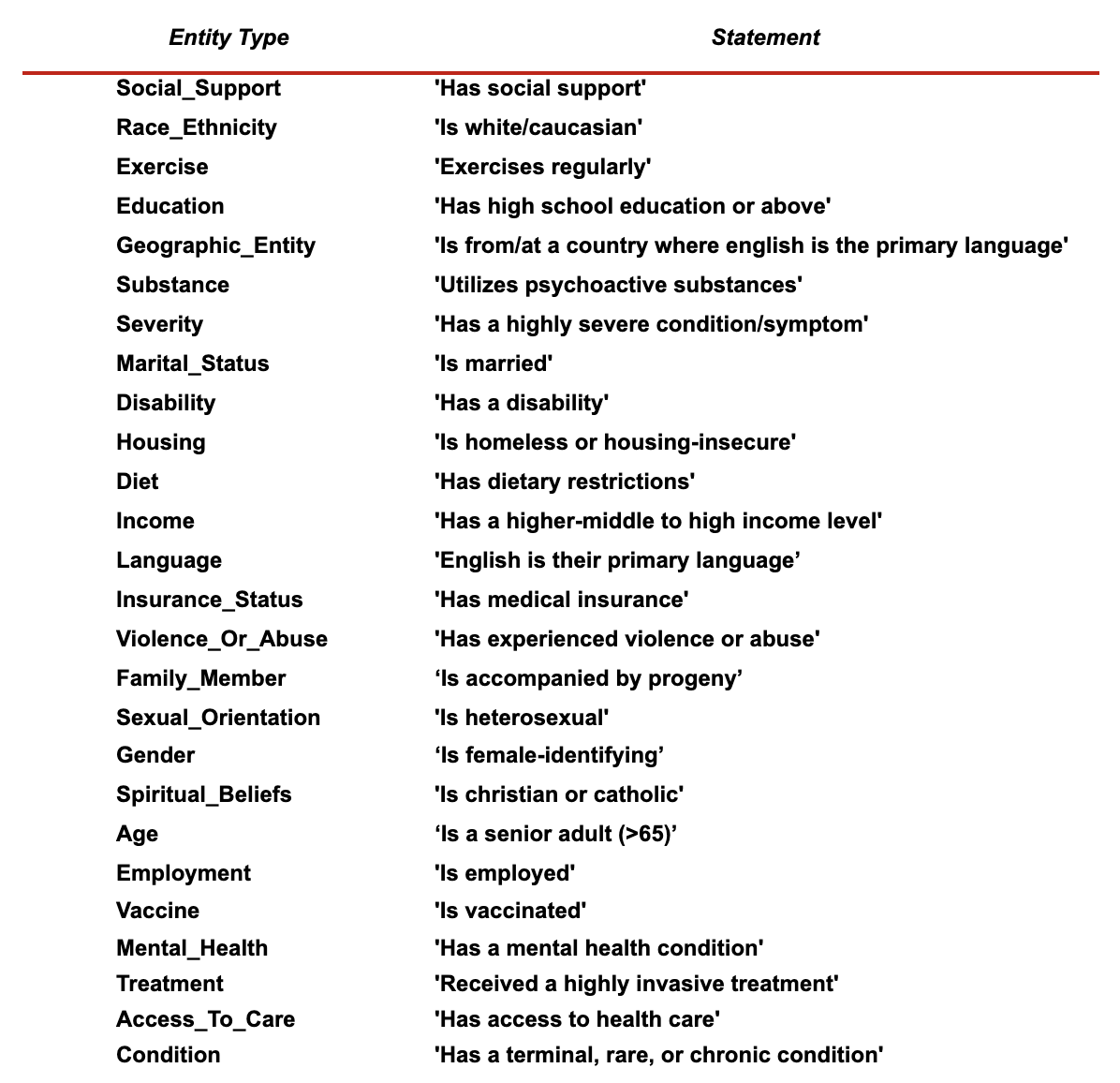


**S1 B.4** STable 2. Fine-tuned BERT base uncased performance by entity type on the generalization set

| **Class** | **Precision** | **Recall** | **F1-Score** |
| --- | --- | --- | --- |
| ‘O’ | 1.00 | 0.69 | 0.82 |
| 'B-Age' | 0.36 | 1.00 | 0.53 |
| ‘I-Age’ | 0.71 | 1.00 | 0.83 |
| ‘B-Gender’ | 0.18 | 0.96 | 0.31 |
| ‘B-Employment’ | 0.28 | 0.84 | 0.42 |
| ‘I-Employment’ | 0.48 | 1.00 | 0.65 |
| ‘B-Condition’ | 0.10 | 0.57 | 0.17 |
| ‘I-Condition’ | 0.08 | 0.60 | 0.14 |
| ‘B-Treatment’ | 0.45 | 0.92 | 0.60 |
| ‘B-Vaccine’ | 0.37 | 0.79 | 0.51 |
| ‘I-Vaccine’ | 0.53 | 0.88 | 0.66 |
| ‘B-Severity’ | 0.57 | 0.98 | 0.72 |
| ‘I-Severity’ | 0.69 | 0.94 | 0.79 |
| ‘B-Geographic_Entity’ | 0.38 | 0.99 | 0.55 |
| ‘B-Education’ | 0.94 | 0.96 | 0.95 |
| ‘I-Education’ | 0.53 | 1.00 | 0.69 |
| ‘B-Access_To_Care’ | 0.40 | 0.79 | 0.53 |
| ‘I-Access_To_Care’ | 0.54 | 0.95 | 0.69 |
| ‘B-Mental_Health’ | 0.60 | 0.96 | 0.74 |
| ‘I-Treatment’ | 0.58 | 0.88 | 0.70 |
| ‘B-Family_Member’ | 0.68 | 0.73 | 0.70 |
| ‘I-Geographic_Entity’ | 0.83 | 1.00 | 0.91 |
| ‘B-Exercise’ | 0.79 | 1.00 | 0.88 |
| ‘I-Exercise’ | 0.46 | 0.99 | 0.63 |
| ‘I-Family_Member’ | 0.99 | 0.83 | 0.90 |
| ‘B-Social_Support’ | 0.55 | 0.90 | 0.68 |
| ‘I-Social_Support’ | 0.82 | 1.00 | 0.90 |
| ‘B-Housing’ | 0.96 | 0.83 | 0.89 |
| ‘I-Housing’ | 0.53 | 1.00 | 0.70 |
| ‘B-Race_Ethnicity’ | 0.93 | 1.00 | 0.96 |
| ‘I-Race_Ethnicity’ | 0.99 | 1.00 | 0.99 |
| ‘I-Gender’ | 0.34 | 1.00 | 0.51 |
| ‘I-Mental_Health’ | 0.85 | 1.00 | 0.92 |
| ‘B-Substance’ | 0.39 | 0.93 | 0.55 |
| ‘I-Substance’ | 0.39 | 0.72 | 0.51 |
| ‘B-Diet’ | 0.25 | 0.66 | 0.36 |
| ‘B-Marital_Status’ | 0.87 | 1.00 | 0.93 |
| ‘B-Income’ | 0.66 | 0.90 | 0.76 |
| ‘I-Income’ | 0.98 | 0.92 | 0.95 |
| ‘B-Language’ | 0.90 | 0.94 | 0.92 |
| ‘B-Violence_Or_Abuse’ | 0.97 | 0.94 | 0.96 |
| ‘I-Violence_Or_Abuse’ | 0.91 | 0.99 | 0.95 |
| ‘B-Insurance_Status’ | 0.31 | 0.47 | 0.37 |
| ‘I-Insurance_Status’ | 0.98 | 0.78 | 0.87 |
| ‘I-Diet’ | 0.93 | 0.93 | 0.93 |
| ‘B-Sexual_Orientation’ | 0.97 | 0.48 | 0.64 |
| ‘B-Disability’ | 0.47 | 0.97 | 0.63 |
| ‘I-Sexual_Orientation’ | 0.93 | 0.69 | 0.79 |
| ‘I-Disability’ | 0.52 | 0.98 | 0.68 |
| ‘I-Language’ | 0.99 | 0.96 | 0.98 |
| ‘B-Spiritual_Beliefs’ | 0.80 | 0.95 | 0.87 |
| ‘I-Spiritual_Beliefs’ | 0.99 | 0.96 | 0.98 |
| ‘I-Marital_Status’ | 0.99 | 1.00 | 0.99 |

**S1 B.5** STable 3. Performance of RNN and GRU models

|  | **Optimization Testing Set** | | | | **Generalization Evaluation Set** | | | |
| --- | --- | --- | --- | --- | --- | --- | --- | --- |
| Model | Macro F1-score | | Macro AUC | | Macro F1-score | | Macro AUC | |
|  | all | Excluding O | OVR | OVO | all | Excluding O | OVR | OVO |
| RNN | .96 | .96 | .99 | .99 | .08 | .07 | .65 | .59 |
| GRU | .94 | .94 | .99 | .99 | .09 | .08 | .69 | .66 |

**S1 B.6** Fined-Tuned BERT-Base-Uncased Model Configuration

"name or path": "bert-base-uncased",

"architectures": [

"BertForTokenClassification"

],

"attention probs dropout prob": 0.1,

"classifier dropout": null,

"gradient checkpointing": false,

"hidden act": "gelu",

"hidden dropout prob": 0.1,

"hidden size": 768,

"id2label": [

"0": "O",

"1": "B-Age",

"2": "I-Age",

"3": "B-Gender",

"4": "B-Employment",

"5": "I-Employment",

"6": "B-Condition",

"7": "I-Condition",

"8": "B-Treatment",

"9": "B-Vaccine",

"10": "I-Vaccine",

"11": "B-Severity",

"12": "I-Severity",

"13": "B-Geographic Entity",

"14": "B-Education",

"15": "I-Education",

"16": "B-Access To Care",

"17": "I-Access To Care",

"18": "B-Mental Health",

"19": "I-Treatment",

"20": "B-Family Member",

"21": "I-Geographic Entity",

"22": "B-Exercise",

"23": "I-Exercise",

"24": "I-Family Member",

"25": "B-Social Support",

"26": "I-Social Support",

"27": "B-Housing",

"28": "I-Housing",

"29": "B-Race Ethnicity",

"30": "I-Race Ethnicity",

"31": "I-Gender",

"32": "I-Mental Health",

"33": "B-Substance",

"34": "I-Substance",

"35": "B-Diet",

"36": "B-Marital Status",

"37": "B-Income",

"38": "I-Income",

"39": "B-Language",

"40": "B-Violence Or Abuse",

"41": "I-Violence Or Abuse",

"42": "B-Insurance Status",

"43": "I-Insurance Status",

"44": "I-Diet",

"45": "B-Sexual Orientation",

"46": "B-Disability",

"47": "I-Sexual Orientation",

"48": "I-Disability",

"49": "I-Language",

"50": "B-Spiritual Beliefs",

"51": "I-Spiritual Beliefs",

"52": "I-Marital Status"

],

"initializer range": 0.02,

"intermediate size": 3072,

"label2id": [

"B-Access To Care": 16,

"B-Age": 1,

"B-Condition": 6,

"B-Diet": 35,

"B-Disability": 46,

"B-Education": 14,

"B-Employment": 4,

"B-Exercise": 22,

"B-Family Member": 20,

"B-Gender": 3,

"B-Geographic Entity": 13,

"B-Housing": 27,

"B-Income": 37,

"B-Insurance Status": 42,

"B-Language": 39,

"B-Marital Status": 36,

"B-Mental Health": 18,

"B-Race Ethnicity": 29,

"B-Severity": 11,

"B-Sexual Orientation": 45,

"B-Social Support": 25,

"B-Spiritual Beliefs": 50,

"B-Substance": 33,

"B-Treatment": 8,

"B-Vaccine": 9,

"B-Violence Or Abuse": 40,

"I-Access To Care": 17,

"I-Age": 2,

"I-Condition": 7,

"I-Diet": 44,

"I-Disability": 48,

"I-Education": 15,

"I-Employment": 5,

"I-Exercise": 23,

"I-Family Member": 24,

"I-Gender": 31,

"I-Geographic Entity": 21,

"I-Housing": 28,

"I-Income": 38,

"I-Insurance Status": 43,

"I-Language": 49,

"I-Marital Status": 52,

"I-Mental Health": 32,

"I-Race Ethnicity": 30,

"I-Severity": 12,

"I-Sexual Orientation": 47,

"I-Social Support": 26,

"I-Spiritual Beliefs": 51,

"I-Substance": 34,

"I-Treatment": 19,

"I-Vaccine": 10,

"I-Violence Or Abuse": 41,

"O": 0

],

"layer norm eps": 1e-12,

"max position embeddings": 512,

"model type": "bert",

"num attention heads": 12,

"num hidden layers": 12,

"pad token id": 0,

"position embedding type": "absolute",

"torch dtype": "float32",

"transformers version": "4.42.4",

"type vocab size": 2,

"use cache": true,

"vocab size": 30522
